# A randomized controlled trial of personalized text messages for smoking cessation, China

**DOI:** 10.2471/BLT.22.289051

**Published:** 2023-02-01

**Authors:** Haoxiang Lin, Xiaoyuan Li, Yanzhen Zhang, Zhe Wen, Zhiyong Guo, Yang Yang, Chun Chang

**Affiliations:** a Institute for Global Health and Development, Peking University, Beijing, China.; b Baotou Patriotic Health Guidance Center, Baotou, China.; c Qingshan District Health Committee, Baotou, China.; d Department of Social Medicine and Health Education, School of Public Health, Peking University, 38 Xueyuan Rd, Haidian District, Beijing, China.

## Abstract

**Objective:**

To describe a tobacco cessation intervention using personalized mobile phone text messages based on behaviour change theory and to assess why the intervention was effective.

**Methods:**

We conducted a two-arm, double-blind, randomized controlled trial in five cities in China from April to July 2021. We recruited daily or weekly smokers aged 18 years or older. The 90-day intervention was delivered using a mobile phone chat application. At different stages of quitting, intervention group participants received personalized text messages based on analyses of the strength of their intention to quit, their motivation to quit and their self-reported success at quitting. Control group participants received non-personalized text messages. The primary outcome was the biochemically verified 6-month abstinence rate. Secondary outcomes were changes in scores on the components of protection motivation theory. All analyses were by intention to treat.

**Findings:**

We randomly assigned 722 participants to intervention or control groups. Biochemically verified continuous abstinence at 6 months was 6.9% (25/360) in the intervention group and 3.0% (11/362) in the control group. Smokers who received the personalized intervention had lower scores on intrinsic rewards of smoking and response costs of quitting in the protection motivation theory analysis. These two variables were also determinants of sustained abstinence, thus explaining why the intervention group had a higher quitting rate.

**Conclusion:**

The study confirmed the psychological determinants of long-term abstinence from smoking and provided a framework to explore why such an intervention is effective. This approach may be applicable to the development or analysis of interventions targeting other health behaviours.

## Résumé

**Objectif:**

Présenter une intervention de sevrage tabagique articulée autour de messages SMS envoyés sur les téléphones mobiles et personnalisés en fonction de la théorie du changement de comportement, puis déterminer pourquoi cette intervention a porté ses fruits.

**Méthodes:**

Nous avons mené un essai contrôlé randomisé en double aveugle et à deux volets dans cinq villes de Chine entre avril et juillet 2021. Nous avons recruté des fumeurs quotidiens ou hebdomadaires âgés de 18 ans et plus. L'intervention, qui s'étalait sur 90 jours, s'est déroulée par l'intermédiaire d'une application de chat sur téléphone mobile. À différents stades du sevrage, les participants du groupe d'intervention ont reçu des messages SMS personnalisés basés sur une analyse de leur degré de volonté et de leur motivation à arrêter de fumer, mais aussi de leurs capacités autodéclarées à y parvenir. De leur côté, les participants du groupe de contrôle ont reçu des messages SMS non personnalisés. Le taux d'abstinence à six mois vérifié par test biochimique constituait le critère d'évaluation principal. Les critères d'évaluation secondaires étaient l'évolution des scores au niveau des composantes de la théorie de la motivation à la protection. Toutes les analyses reposaient sur l'intention de traiter.

**Résultats:**

Nous avons réparti aléatoirement 722 participants entre groupes d'intervention et de contrôle. Le taux d'abstinence à six mois vérifié par test biochimique s'élevait à 6,9% (25/360) au sein du groupe d'intervention et à 3,0% (11/362) au sein du groupe de contrôle. Dans le cadre de l'analyse fondée sur la théorie de la motivation à la protection, les fumeurs ayant fait l'objet d'une action personnalisée ont obtenu des scores plus faibles en matière de récompense intrinsèque provoquée par le tabagisme et de coûts d'intervention du sevrage. Ces deux variables se sont également révélées décisives dans l'abstinence prolongée, ce qui explique pourquoi le groupe d'intervention a affiché un taux de sevrage plus conséquent.

**Conclusion:**

L'étude a confirmé l'impact des déterminants psychologiques de l'abstinence à long terme et fourni des pistes d'exploration des raisons pour lesquelles une telle action est efficace. Cette approche pourrait être utilisée dans le développement ou l'analyse d'interventions ciblant d'autres comportements liés à la santé.

## Resumen

**Objetivo:**

Describir una intervención para dejar de fumar mediante el uso de mensajes de texto personalizados para teléfonos móviles basada en la teoría del cambio de conducta y evaluar por qué la intervención fue eficaz.

**Métodos:**

Se realizó un ensayo aleatorizado, con enmascaramiento doble y con dos grupos en cinco ciudades de China entre abril y julio de 2021. Se preseleccionaron fumadores diarios o semanales mayores de 18 años. La intervención de 90 días se realizó mediante una aplicación de chat para teléfonos móviles. En las diferentes etapas de abstinencia, los participantes del grupo de intervención recibieron mensajes de texto personalizados según los análisis de la fuerza de su intención de dejar de fumar, su motivación para dejar de fumar y su éxito autoinformado al dejar de fumar. Los participantes del grupo comparativo recibieron mensajes de texto no personalizados. El criterio de valoración principal fue la tasa de abstinencia verificada mediante pruebas bioquímicas a los 6 meses. Los criterios de valoración secundarios fueron los cambios en las puntuaciones de los componentes de la teoría de la motivación de protección. Todos los análisis se realizaron por intención de tratar.

**Resultados:**

Se asignaron aleatoriamente 722 participantes a los grupos de intervención o comparativos. La abstinencia continua verificada mediante pruebas bioquímicas a los 6 meses fue del 6,9 % (25/360) en el grupo de intervención y del 3,0 % (11/362) en el grupo comparativo. Los fumadores que recibieron la intervención personalizada tuvieron puntuaciones más bajas en las recompensas intrínsecas de fumar y en los costes de respuesta de dejar de fumar en el análisis de la teoría de la motivación de protección. Estas dos variables también fueron determinantes de la abstinencia prolongada, lo que explica por qué el grupo de intervención tuvo una tasa de abstinencia más elevada.

**Conclusión:**

El estudio confirmó los determinantes psicológicos de la abstinencia del tabaco a largo plazo y proporcionó un marco para explorar por qué una intervención de este tipo es eficaz. Este enfoque podría aplicarse al desarrollo o análisis de intervenciones dirigidas a otros hábitos saludables.

## ملخص

**الغرض:**

وصف التدخل للإقلاع عن تعاطي التبغ باستخدام رسائل نصية مخصصة للهاتف المحمول بناءً على نظرية تغيير السلوك، وتقييم سبب فعالية التدخل.

**الطريقة:**

قمنا بإجراء تجربة تحكم عشوائية، ثنائية التعمية، ثنائية الذراع، في خمس مدن في الصين في الفترة من أبريل/نيسان إلى يوليو/تموز 2021. قمنا بتوظيف مدخنين يوميًا أو أسبوعيًا بعمر 18 عامًا أو أكبر. تم تنفيذ التدخل لمدة 90 يومًا باستخدام تطبيق للدردشة على الهاتف المحمول. في مراحل مختلفة من الإقلاع، تلقى المشاركون في مجموعة التدخل رسائل نصية مخصصة بناءً على تحليلات شدة عزمهم على الإقلاع، ودوافعهم للإقلاع، ونجاحهم المبلغ عنه ذاتيًا في الإقلاع. تلقى المشاركون في مجموعة التحكم رسائل نصية غير مخصصة. كانت النتيجة الأولية هي معدل الامتناع عن ممارسة الجنس لمدة 6 أشهر، والذي تم التحقق منه كيميائياً. كانت النتائج الثانوية هي تغييرات في الدرجات على مكونات نظرية دافع الحماية. كانت جميع التحليلات مرتكزة على نية العلاج.

**النتائج:**

قمنا بتعيين 722 مشاركًا بشكل عشوائي لمجموعات التدخل أو مجموعات التحكم. كان الامتناع المتواصل عن ممارسة الجنس لمدة 6 أشهر، والذي تم التحقق منه كيميائيا، عند معدل %6.9 (25/360) في مجموعة التدخل، ومعدل %3.0 (11/362) في مجموعة التحكم. المدخنون الذين تلقوا التدخل الشخصي حصلوا على درجات أقل في المكافآت الجوهرية للتدخين وتكاليف الاستجابة للإقلاع، في تحليل نظرية دوافع الحماية. كان هذان المتغيران أيضًا من العوامل المحددة للامتناع المتواصل عن ممارسة الجنس، وبذلك يفسر سبب ارتفاع معدل الإقلاع عن التدخين لدى مجموعة التدخل.

**الاستنتاج:**

أكدت الدراسة المحددات النفسية للامتناع طويل المدى عن التدخين، وقدمت إطار عمل لاستكشاف سبب فعالية مثل هذا التدخل. قد يكون هذا النهج قابلاً للتطبيق لتطوير أو تحليل التدخلات التي تستهدف السلوكيات الصحية الأخرى.

## 摘要

**目的:**

描述基于行为改变理论的个性化手机短信戒烟干预，并评估干预有效的原因。

**方法:**

我们于 2021 年 4 月至 7 月在中国五个城市进行了一项双臂双盲随机对照试验。我们招募了年龄为 18 岁或以上且每日或每周吸烟的人。使用手机聊天应用程序进行为期 90 天的干预。在戒烟的不同阶段，干预组的研究对象会收到个性化的短信，这些短信是根据对他们的戒烟意愿强度、戒烟动机和自我报告的戒烟成功情况的分析编制的。对照组的研究对象会收到非个性化的短信。主要结局指标是经生化指标验证的 6 个月持续戒烟率。次要结局指标是保护动机理论各组成部分的分数变化。所有分析均按照意向性治疗原则。

**结果:**

我们将 722 名参与者随机分配到干预组或对照组。干预组经生化指标验证的6 个月持续戒烟率为 6.9% (25/360)，对照组为 3.0% (11/362)。在保护动机理论分析中，接受个性化干预的吸烟者在吸烟的内部回报和戒烟的反应代价方面得分较低。这两个变量也是持续戒烟的决定因素，从而解释了干预组戒烟率较高的原因。

**结论:**

该研究证实了长期戒烟的心理决定因素，并为探索为什么这种干预有效提供了一个框架。这种方法可能适用于针对其他健康行为的干预措施的开发或分析。

## Резюме

**Цель:**

Описать меры по прекращению употребления табака с использованием персонализированных текстовых сообщений для мобильного телефона, основанные на теории изменения моделей поведения, и оценить причины эффективности этих мер.

**Методы:**

В период с апреля по июль 2021 года в пяти городах Китая было проведено двойное слепое рандомизированное контролируемое исследование в двух группах. Для исследования были отобраны курильщики в возрасте 18 лет и старше, курящие ежедневно или еженедельно. На протяжении 90 дней проводились мероприятия с использованием приложения для чата на мобильном телефоне. На разных этапах отказа от курения участники экспериментальной группы получали персонализированные текстовые сообщения, основанные на анализе силы их намерения отказаться от курения, их мотивации отказаться от курения и отмеченных самими участниками успехов в отказе от курения. Участники контрольной группы получали неперсонализированные текстовые сообщения. Первичным исходом был биохимически подтвержденный показатель отказа от курения в течение 6 месяцев. Вторичными исходами были изменения в баллах по компонентам теории мотивации защиты. Все анализы проводили по всем пациентам, рандомизированным для участия в исследовании.

**Результаты:**

722 участника были случайным образом распределены в экспериментальную или контрольную группу. Биохимически подтвержденный показатель непрерывного отказа от курения через 6 месяцев составил 6,9% (25 из 360) в экспериментальной группе и 3,0% (11 из 362) в контрольной группе. У курильщиков, получивших персонализированные меры воздействия, наблюдались более низкие показатели по внутреннему вознаграждению за курение и реакции на отказ от курения при анализе теории мотивации защиты. Эти две переменные также определяли продолжительный отказ от курения, что объясняет причину более высокого показателя отказа от курения в экспериментальной группе.

**Вывод:**

В ходе исследования были подтверждены психологические детерминанты долгосрочного отказа от курения и создана основа для изучения причин эффективности подобных мер воздействия. Этот подход может быть применим к разработке или анализу мер воздействия, направленных на другие модели поведения в отношении здоровья.

## Introduction

Tobacco use is still a major public health problem worldwide. The most common type of tobacco use is cigarette smoking, which is one of the leading preventable causes of death, responsible for more than 600 million deaths globally every year. Available data suggest that tobacco users lose 15 years of life and live with functional disabilities. Therefore, helping people stop using tobacco is the most cost–effective public health intervention.^1^

Smoking cessation services are a critical part of the intervention. However, providing universal smoking cessation support can be a challenge for health services in most low- and middle-income countries, as it requires sufficient resources.^2^^,^^3^ One way to expand access is to use mobile phone-based technology to provide cessation support.^4^

Many studies have shown that mobile phones have the potential to provide smoking cessation support. One previous randomized trial published in *The Lancet* showed that biochemically verified continuous abstinence at 6 months was significantly increased in a text messaging group compared with a blank control group (10.7% versus 4.9%).^5^ A more recent study found that the rate of biochemically verified continuous abstinence at 24 weeks was 6.5% in the high-frequency messaging group, 6.0% in the low-frequency messaging group and 1.9% in the control group.^6^

Although mobile phone-based tobacco cessation initiatives have generated considerable enthusiasm,^5^^–^^7^ previous studies leave important questions unanswered. First, most of these studies found that such interventions have a higher quitting rate than interventions providing minimal support for smoking cessation.^5^^,^^6^ However, why such interventions are effective has been less well studied. As a result, once the effectiveness of a mobile phone-based cessation intervention has been tested, researchers are unsure how to adjust or scale up the intervention. Second, most current mobile phone-based cessation studies have limited descriptions of the intervention principles, which leaves researchers unclear as to how they can take advantage of previous experiences.^8^

To help fill the evidence gap, we conducted a randomized, controlled trial of a personalized mobile phone-based smoking cessation intervention in China. We aimed to assess why such an intervention is effective and to provide an analysis framework to explore the reasons behind the quitting rate. The study extends the previous research in two ways. First, the intervention used personalized text messages in a country with a limited tobacco control policy. Second, we go beyond simply determining the quitting rate to study the psychological determinants of successful quitting and quitting behaviours.

## Methods

### Study design

We conducted a two-arm, double-blind, randomized controlled trial in five cities in China (Beijing, Baotou, Dezhou, Linzi and Yakeshi). The locations of the study provinces are shown in the author’s online repository.^9^ Participants were randomized to intervention or control groups between April 2021 and July 2021. We used the WeChat application, the most popular Chinese online chatting application (Tencent Holdings Limited, Wanchai, China, Hong Kong Special Administrative Region) to collect and analyse data from participants and to deliver smoking cessation messages to participants on their mobile phones. 

The trial was approved by the ethics committeee of Peking University Health Science Center (IRB00001052–30063). All the participants signed informed consent forms before randomization and knew that they could withdraw from the study at any time. All patients’ information was accessible only by the personnel participating in the study. We did not provide money to the participants, but we provided gifts (a towel, an umbrella or a cup) if the participants completed one follow-up visit. The clinical trial registration number is ChiCTR2100041942. This study has no changes from the original proposal in terms of methods or outcome measures after the trial began.

### Theoretical framework

The theoretical framework of the intervention was based on the transtheoretical model of behaviour change and the protection motivation theory. Both models have been independently applied to study health behaviour change interventions.^10^^,^^11^

The transtheoretical model proposes a systematic relationship between the stages and processes of behaviour change. To apply the model, we therefore used several strategies to strengthen change (in this case, quitting smoking) or to achieve the next stage of change. To prepare smokers for quitting, we formulated messages based on an analysis of smokers’ quitting intention. Messages for smokers with weak quitting intention were based on consciousness raising, dramatic relief and environmental re-evaluation (see Box 1 for an explanation of the terms). Messages for smokers with strong quitting intention were framed around stimulus control, self-liberation and reinforcement management. To encourage smokers who relapsed from their quitting attempt, we formulated messages based on consciousness raising, dramatic relief, environmental re-evaluation and self-re-evaluation.

Box 1 **Explanation of the components of two models used in the study of a personalized smoking cessation intervention, China**

***Transtheoretical model of behaviour change***

Consciousness raising: finding and learning new facts, ideas and tips that support the healthy behavioural change.

Dramatic relief: experiencing the negative emotions (fear, anxiety, worry) that go along with unhealthy behavioural risks.

Environmental re-evaluation: realizing the negative impact of the unhealthy behaviour or the positive impact of the healthy behaviour on one’s social and physical environment.

Self-re-evaluation: realizing that the behavioural change is an important part of one’s identity as a person.

***Protection motivation theory***

Perceived severity: beliefs about the negative consequences of the health threat.

Perceived vulnerability: vulnerability to the negative consequences of the threatened event.

Intrinsic and extrinsic rewards: the benefits of the performance of the maladaptive behaviour.

Self-efficacy: confidence in one’s ability to perform the preventive behaviour.

Response efficacy: beliefs about the effectiveness of the preventive behaviour for the threatened event.

Response cost: barriers to performance of the preventive behaviour.

For smokers who remained abstinent, messages were only based on protection motivation theory. The protection motivation theory has seven components (sub-constructs) which we applied to study the psychological determinants of participants’ smoking behaviour: perceived severity of smoking-related diseases; perceived vulnerability to smoking-related diseases; intrinsic rewards of smoking; extrinsic rewards of smoking; self-efficacy of quitting; response efficacy of quitting; and response cost of quitting.

### Intervention

A detailed framework of the intervention based on the two behaviour change models is shown in Table 1. The total intervention lasted for 3 months. Our team wrote special modules for the WeChat application to collect and analyse data and deliver messages. 

**Table 1:** Theoretical framework for the study of a personalized smoking cessation intervention, China

Programme phase	Transtheoretical model	Protection motivation theory	Message bank	Example text message	Personalized information
Day 0: Registration	NA	NA	NA	In the next three months, we will support you to quit smoking. Please pay attention to the information we send through WeChat. [DATE] will be your quit day. After that day, you should not even try a cigarette. You still have a few days to prepare.	No
Day 1–7: Pre-quitting phase (P)	*Weak quitting intention (W):* Consciousness raising Dramatic relief Environmental re-evaluation	Increased severity and susceptibility (SS)	P-W and P-SS	P-W: Your children may imitate your smoking behaviour; quitting smoking will set a good example for your family. P-S: Heavy work or lack of sleep lead to relapse; we encourage you to better arrange your time in the early stage of quitting. P-SS: 50%of smokers will die early from smoking. P-RR: Quitting smoking is actually the most cost–effective health care measure. P-EE: If there is a smoker in your home, you can encourage him or her to quit smoking together with you.	Yes
		Decreased response cost, intrinsic and extrinsic rewards (RR)	P-W and P-RR		Yes
		Increased self-efficacy and response efficacy (EE)	P-W and P-EE		Yes
	*Strong quitting intention (S):* Stimulus control Self-liberation Reinforcement management	Increased severity and susceptibility (SS)	P-S and P-SS		Yes
		Decreased response cost, intrinsic and extrinsic rewards (RR)	P-S and P-RR		Yes
		Increased self-efficacy and response efficacy (EE)	P-S and P-EE		Yes
Day 8: Quit day	NA	NA	NA	Congratulations! Today is your quit day. 1. Try not to smoke after today, not even a puff. 2. Please remove all the tobacco products from your possession. 3. Avoid drinking alcohol or attending parties with smokers.	No
Day 9–18: Withdrawal symptom management	NA	NA	NA	Please get enough sleep; you may take a nap, perform moderate exercise, take a hot bath and drink more water.	No
Day 19–36: Early quitting phase (E)	*Relapsed (R):* Consciousness raising Dramatic relief Environmental re-evaluation Self-re-evaluation	Increased severity and susceptibility (SS)	E-R and E-SS	E-R: You can choose alternatives to help overcome the urge to smoke, such as using gum or a toothpick to keep your mouth busy. E-SS: Compared with smoking regular cigarettes, smoking low-tar cigarettes does not reduce the harm of smoking. E-RR: Currently, everyone is pursuing a healthy lifestyle; smoking is no longer a social trend. E-EE: Your efforts are for a better life; many people like you are quitting smoking with us now	Yes
		Decreased response cost, intrinsic and extrinsic rewards (RR)	E-R and E-RR		Yes
		Increased self-efficacy and response efficacy (EE)	E-R and E-EE		Yes
	*Maintained abstinence:* No information from the transtheoretical model	Increased severity and susceptibility (SS)	E-SS		Yes
		Decreased response cost, intrinsic and extrinsic rewards (RR)	E-RR		Yes
		Increased self-efficacy and response efficacy (EE)	E-EE		Yes
Day 37–90: Late quitting phase (L)	*Relapsed (R):* Consciousness raising Dramatic relief Environmental re-evaluation Self-re-evaluation	Increased severity and susceptibility (SS)	L-R and L-SS	L-R: After you maintain abstinence for a period of time, you will feel refreshed and energetic. Keep trying, you will feel it soon. L-SS: Even occasional smoking can cause serious health problems; therefore, we need to quit smoking, not reduce the smoking amount. L-RR: Quitting smoking will not cause any problems to your body; the possible discomfort in the early weeks of quitting is called withdrawal symptoms, which can disappear after a few weeks. L-EE: You may have noticed some improvements in your body, such as increased appetite and easy breathing, which are the benefits of quitting smoking.	Yes
		Decreased response cost, intrinsic and extrinsic rewards (RR)	L-R and L-RR		Yes
		Increased self-efficacy and response efficacy (EE)	L-R and L-EE		Yes
	*Maintained abstinence:* No information from the transtheoretical model	Increased severity and susceptibility (SS)	L-SS		Yes
		Decreased response cost, intrinsic and extrinsic rewards (RR)	L-RR		Yes
		Increased self-efficacy and response efficacy (EE)	L-EE		Yes

NA: not applicable.

We designed a message bank for WeChat with a three-layer framework. The first layer was divided based on the timing of messages and consisted of the pre-quitting message (days 1–7), quitting day message (day 8), withdrawal symptom management message (days 9–18), early quit phase message (days 19–36) and late quit phase message (days 37–90). The second layer was divided based on the transtheoretical model. Before the quit day, messages were classified as: strong quitting intention or weak quitting intention. After the quit day, messages were classified as: maintained abstinence or relapsed. The third layer was divided based on the protection motivation theory. Because of some overlap among the seven components of the protection motivation theory, we merged them into three groups: increased severity and susceptibility; decreased response cost and intrinsic and extrinsic rewards; or increased self-efficacy and response efficacy. The core motivational messages consisted of 14 sub-groups with a total of 200 text messages. There were also approximately 200 contact messages. The intervention group participants received partial information, and the information they received varied depending on their smoking status and scores on the protection motivation theory components.

The WeChat application calculated each participant’s score in the protection motivation theory by delivering questions and recording information. The programme then automatically calculated the lower score of the components of the model that needed to be strengthened. Specifically, the scale comprised 21 items using a 7-point Likert-type scale with responses ranging from 1 (definitely disagree) to 7 (definitely agree). Each construct of protection motivation theory included three items, and we computed the mean of the sub-scale score. We have published the details of this scale and evaluation process elsewhere.^12^^,^^13^ The WeChat application determined each smoker’s quitting intention by using a 5-point scale ranging from not at all likely (score 1), to very likely (score 5), to in the likelihood that they would try to quit in the next 6 months.^14^

### Implementation

#### Participants

The sample size calculation for the study was based on the formula for a two-arm randomized controlled trial. Based on earlier research, we estimated that biochemically verified continuous smoking abstinence at 6 months would be approximately 4% in the control group and 10% in the intervention groups.^5^^,^^6^^,^^15^ To achieve 80% power with a significance level of 0.05 (two-sided), a sample size of 280 individuals was needed in each group. Assuming 20% attrition in the follow-up measurements, the total required sample size was 672.

We advertised the trial to smokers through leaflets, digital advertisements on WeChat and via teachers and community leaders. Potential participants contacted the local disease control and prevention centre to register. Daily or weekly smokers aged 18 years or older were eligible for inclusion if they owned a mobile phone and used the WeChat application. All eligible smokers were told they needed to come to a specific place on a fixed date to finalize the recruitment process. The eligibility of participants was double-checked by the research team or local centre staff, and participants signed an informed consent form at the first face-to-face contact.

#### Procedure

After recruitment, participants were required to complete the baseline questionnaire and register through WeChat. We first classified our participants into two groups: low nicotine dependence; or moderate and high nicotine dependence. Then we used a simple randomization method to assign participants to the intervention group or control group within each nicotine dependence group.^16^ We also used the WeChat programme to balance the demographic characteristics of participants in the two groups. Randomization was fully computerized and automated with equal allocation. The researchers and participants were all blind to the group allocation.

All participants in the two groups were instructed to attend face-to-face follow-ups with research staff at 1 month, 3 months and 6 months after randomization. At each follow-up visit for all participants, we recorded smoking status, score on the protection motivation theory components and quitting intention.

Participants who were allocated to the intervention group received the intervention programme with personalized text messages, as described above. The information that participants received from the WeChat message bank varied depending on the evaluation of their motivation to quit and their self-reported smoking status. On day 0, all the intervention participants received the same message about registration in the programme and completed the first evaluation via the chat application (Table 1). Days 1–7 were the pre-quitting phase. Participants received two personalized messages per day: one message based on their intention to quit (strong or weak) and one message based on their motivation to quit, as described earlier (severity and susceptibility; response-cost, intrinsic and extrinsic rewards; or self-efficacy and response efficacy). Day 8 was the proposed quitting day. Messages on this day were reminders to stop smoking and preparations for quitting. From day 9–18, participants received one message per day related to management of withdrawal symptoms. From days 19–36, all the messages were selected from the early quitting phase message bank. Participants received two messages per day: one message based on their smoking status (relapsed or maintained abstinence) and one message based on their motivation to quit, classified in the same way as in the pre-quitting phase. From days 37–90, all the messages were selected from the late quitting phase message bank. The messages were the same as those sent on days 19–36. The WeChat application analysed smokers’ status on day 0, day 19, day 36, day 45, day 60 and day 75 to select what messages to send them.

Participants in the control group received a set of messages developed by the United States National Cancer Institute, which have been adapted to be culturally appropriate for a Chinese audience.^15^ This message bank was based on well-established cognitive–behavioural cessation approaches and contained 91 messages. The text messages provided encouragement, practical advice to help maintain cessation and information on the health effects of smoking. All control participants received the same text messages on the same days without a personalized evaluation. For the control group, scores on the protection motivation theory components were obtained in face-to-face visits not via the WeChat application.

Both groups received 1–2 messages a day for 3 months after randomization.

#### Outcomes

The primary outcome was the biochemically verified, 6-month sustained abstinence rate, defined as participants’ self-reports of not smoking any cigarettes after the designated quitting date. Self-reported quitting was validated biochemically by an expired carbon monoxide level of less than 6 ppm at each follow-up visit.^17^ Secondary outcomes were the change in scores on the seven components of the protection motivation theory.

### Data analysis

We conducted the data analysis in three steps. First, we used descriptive statistics to report smoking abstinence rates and used *χ^2^* analyses to compare the abstinence rates between the two groups. We performed intention-to-treat analysis for each group. 

Second, we analysed the change in the scores on the protection motivation theory components, by group, to evaluate the effect of our intervention in strengthening awareness about quitting. We used generalized estimated equations to analyse the data with repeated follow-up times.^18^ We estimated the impact of the intervention on participants’ scores on the protection motivation theory in the first set of models. The dependent variables were the seven components of the protection motivation theory. The explanatory variable was the group (intervention group = 1; control group = 0). We added an interaction term in the model with two variables (time and group) to show how the intervention effect changed with time. We controlled for age, education level, urban or rural area, nicotine dependence and alcohol consumption in the analyses. The results are presented as odds ratios (OR) and 95% confidence intervals (CIs).

Third, we analysed the association between participants’ scores on the protection motivation theory components and their continuous abstinence behaviour to explore why the intervention group reported a higher quitting rate. We changed the dependent variable to a dummy variable indicating whether the respondent remained abstinent during the follow-up period (if yes = 1; otherwise = 0). The explanatory variables were the protection motivation theory component scores. We used the second generalized estimated equations to assess which components were associated with sustained abstinence to further explore the reason for the higher sustained abstinence rate in the intervention group. We chose an exchangeable correlation structure for the matrix structure, and binary logistic regression for the model setting. 

We conducted all statistical analyses using SPSS, version 19.0 (Armonk, IBM, United States of America). A *P*-value < 0.05 was considered significant.

## Results

**Fig. 1:**
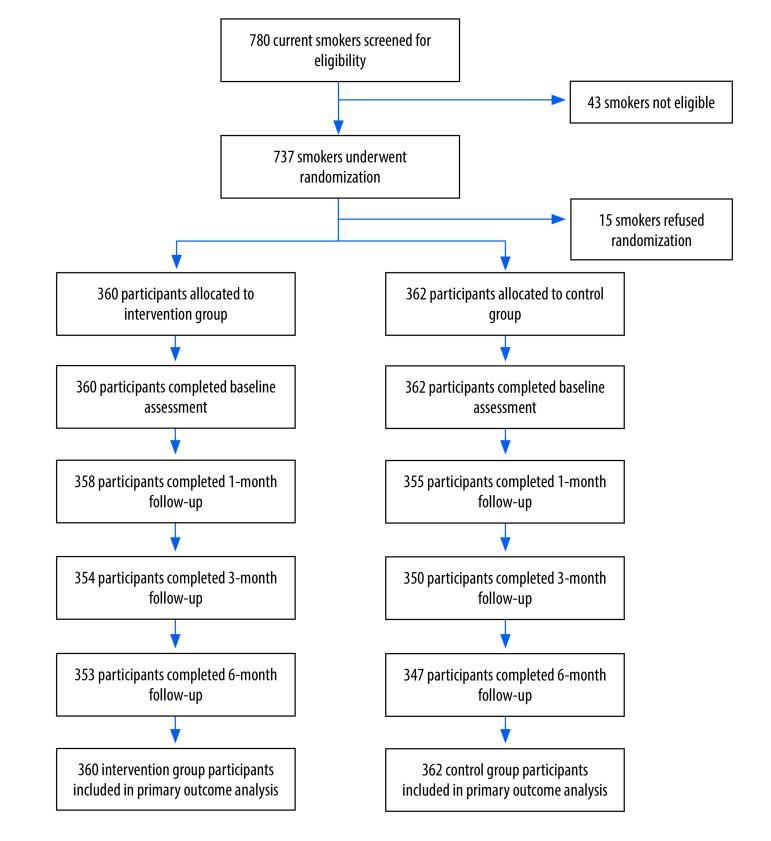
Flowchart of the study of a personalized text message smoking cessation intervention, China

We randomly assigned 722 participants to the intervention or control group (Fig. 1). Treatment groups were well-balanced with respect to baseline characteristics (Table 2). All the loss to follow-up in this study was caused by refusal to connect or reply. According to the Russell standard, those who declined to be involved in subsequent data collection were counted as smokers.^17^ Biochemically verified continuous abstinence at 6 months was 6.9% (25/360 participants) in the intervention group and 3.0% (11/362 participants) in the control group (OR: 2.38; 95% CI: 1.15–4.92).

**Table 2:** Baseline characteristics of participants in the study of a personalized text message smoking cessation intervention, China

Variable	No. (%) of participants			*P*-value^a^
	Control group (*n* = 362)	Intervention group (*n* = 360)	Total	
**Sex**				
Male	358 (98.9)	358 (99.4)	716 (99.2)	
Female	4 (1.1)	2 (0.6)	6 (0.8)	0.42
**Age, years**				
18–44	212 (58.6)	184 (51.1)	396 (54.8)	
45–64	143 (39.5)	168 (46.7)	311 (43.1)	0.13
> 64	7 (1.9)	8 (2.2)	15 (2.1)	
**Education**				
Middle school or lower	54 (14.9)	58 (16.2)	112 (15.5)	
High school	73 (20.2)	96 (26.7)	169 (23.4)	0.07
College or above	235 (64.9)	205 (57.1)	440 (61.0)	
**Ethnicity**				
Han	341 (94.2)	336 (93.3)	677 (93.8)	
Other	21 (5.8)	24 (6.7)	45 (6.2)	0.63
**Residential area**				
Urban	230 (65.9)	223 (62.8)	453 (64.3)	
Rural	119 (34.1)	132 (37.2)	251 (35.7)	0.39
**Smoking status**				
Daily smoker	293 (81.4)	299 (83.1)	592 (82.2)	
Weekly smoker	67 (18.6)	61(16.9)	128 (17.8)	0.56
**Monthly income, yuan**				
< 4 000	177 (48.9)	169 (46.9)	346 (47.9)	
4000–5999	115 (31.8)	116 (32.2)	231 (32.0)	0.84
≥ 6 000	70 (19.3)	75 (20.8)	145 (20.1)	
**Nicotine dependence**				
Low	228 (63.3)	228 (63.9)	456 (63.6)	
Moderate	101 (28.1)	101(28.3)	202 (28.2)	0.93
High	31 (8.6)	28 (7.8)	59 (8.2)	

^a^ We calculate P-values using *χ^2^* test.

Table 3 shows the mean scores on the protection motivation theory components, by group and time after starting quitting. There were significant interactions between time and group for some components (Table 4). The intervention group had significantly lower scores for the intrinsic rewards of smoking (OR: 0.73; 95% CI: 0.56–0.96), the extrinsic rewards of smoking (OR: 0.71; 95% CI: 0.55–0.90) and the response cost of quitting (OR: 0.75; 95% CI: 0.57–0.99). These positive effects were stronger and more significant 3 months after the intervention compared with 1 month and 6 months after the quitting day,

**Table 3:** Participants’ scores on protection motivation theory components, by group and time after starting quitting, in the personalized smoking cessation intervention, China

Group and time after quit day	No. of participants	Mean (SD) scores on the protection motivation theory components						
		Severity	Vulnerability	Extrinsic rewards	Intrinsic rewards	Self-efficacy	Response efficacy	Response cost
**Month 0**								
Control group	360	6.13(1.20)	5.37 (1.47)	3.14 (1.45)	4.63 (1.71)	4.55 (1.66)	5.44(1.38)	3.29(1.62)
Intervention group	360	6.21(1.20)	5.36 (1.47)	3.08 (1.45)	4.63 (1.67)	4.60 (1.60)	5.61(1.39)	3.14(1.55)
**Month 1**								
Control group	352	6.10 (1.25)	5.31 (1.46)	2.84 (1.33)	4.23 (1.61)	4.49 (1.65)	5.45(1.43)	3.12(1.52)
Intervention group	355	6.25 (1.20)	5.34 (1.49)	2.75 (1.35)	4.01 (1.69)	4.67 (1.60)	5.73(1.32)	3.02(1.48)
**Month 3**								
Control group	345	6.07(1.23)	5.20 (1.41)	2.88 (1.35)	4.12 (1.55)	4.55 (1.65)	5.54(1.32)	3.09(1.53)
Intervention group	351	6.30(1.16)	5.29 (1.39)	2.57 (1.37)	3.80 (1.66)	4.78 (1.63)	5.87(1.28)	2.71(1.42)
**Month 6**								
Control group	339	6.08 (1.28)	5.25 (1.43)	2.84 (1.42)	4.05 (1.66)	4.62 (1.58)	5.64(1.30)	2.92(1.55)
Intervention group	351	6.36(1.00)	5.34 (1.37)	2.50 (1.32)	3.84 (1.72)	4.79 (1.64)	5.82(1.29)	2.82(1.56)

SD: standard deviation.

**Table 4:** Association between participants’ scores on protection motivation theory components, by group and time after starting quitting, in the personalized smoking cessation intervention, China

Variable	OR (95% CI) of scores on protection motivation theory components						
	Perceived severity	Perceived vulnerability	Intrinsic rewards	Extrinsic rewards	Self-efficacy	Response efficacy	Response cost
**Group**							
Control	Ref.	Ref.	Ref.	Ref.	Ref.	Ref.	Ref.
Intervention	1.07 (0.90–1.28)	1.01 (0.81–1.24)	1.04 (0.81–1.34)	1.06 (0.85–1.31)	1.00 (0.79–1.27)	1.12 (0.91–1.38)	0.93 (0.73–1.18)
**Time after quit day**							
Month 0	Ref.	Ref.	Ref.	Ref.	Ref.	Ref.	Ref.
Month 1	0.92 (0.79–1.07)	0.92 (0.78–1.07)	0.66 (0.55–0.80)	0.78 (0.66–0.92)	0.94 (0.77–1.15)	1.00 (0.84–1.20)	0.85 (0.70–1.02)
Month 3	0.92 (0.79–1.08)	0.83 (0.71–0.98)	0.61 (0.50–0.74)	0.81 (0.68–0.96)	0.96 (0.79–1.18)	1.07 (0.89–1.28)	0.83 (0.67–1.02)
Month 6	0.96 (0.82–1.12)	0.89 (0.75–1.05)	0.56 (0.46–0.69)	0.79 (0.66–0.95)	1.07 (0.87–1.31)	1.19 (1.00–1.41)	0.71 (0.58–0.86)
**Group x time**							
Control	Ref.	Ref.	Ref.	Ref.	Ref.	Ref.	Ref.
Intervention x month 1	1.13 (0.91–1.39)	1.05 (0.83–1.33)	0.80 (0.61–1.05)	0.91 (0.72–1.15)	1.18 (0.91–1.54)	1.18 (0.93–1.49)	1.02 (0.78–1.32)
Intervention x month 3	1.21 (0.98–1.50)	1.14 (0.91–1.43)	0.73 (0.56–0.96)	0.71 (0.55–0.90)	1.26 (0.95–1.65)	1.25 (0.97–1.61)	0.75 (0.57–0.99)
Intervention x month 6	1.21 (0.98–1.49)	1.09 (0.86–1.39)	0.80 (0.61–1.06)	0.68 (0.53–0.87)	1.15 (0.87–1.53)	1.09 (0.86–1.40)	0.99 (0.75–1.30)

CI: confidence interval; OR: odds ratio; Ref.: Reference group.

Notes: We used generalized estimated equations. The dependent variables were the seven components of the protection motivation theory. The explanatory variable was the group (intervention group = 1; control group = 0). We added the interaction term between time and group to show how the intervention effect changed with time. We controlled for age, education level, urban or rural area, nicotine dependence and alcohol consumption. We chose an exchangeable correlation structure for the matrix structure and linear regression for the model setting.

Table 5 shows that the intrinsic rewards of smoking (OR: 0.81; 95%: 0.72–0.92) and the response cost of quitting (OR: 0.84; 95% CI: 0.73–0.97) were negatively associated with sustained abstinence. The self-efficacy of quitting was significantly positively associated with quitting (OR: 2.12; 95% CI: 1.73–2.59). Changes in scores on the other components were not statistically significant. These two variables were also determinants of sustained abstinence, thus explaining why the intervention group had a higher quitting rate.

**Table 5:** Association between participants’ scores on protection motivation theory components and sustained abstinence from smoking in the personalized smoking cessation intervention, China

Variable	OR (95% CI) of sustained abstinence from smoking
**Protection motivation theory components**	
Perceived severity	1.12 (0.87–1.43)
Perceived vulnerability	1.13 (0.97–1.30)
Extrinsic rewards	1.06 (0.91–1.24)
Intrinsic rewards	0.81 (0.72–0.92)
Self-efficacy	2.12 (1.73–2.59)
Response efficacy	0.92 (0.76–1.11)
Response cost	0.84 (0.73–0.97)
**Control variables**	
Carbon monoxide levels	
Baseline readings	0.82 (0.75–0.89)
Nicotine dependence	
High	Ref.
Moderate	1.33 (0.37–4.86)
Low	0.70 (0.19–2.54)
Residential area	
Rural	Ref.
Urban	0.59 (0.36–0.95)
Education	
College or above	Ref.
High school	0.97 (0.56–1.69)
Middle school or lower	0.83 (0.40–1.74)
Chronic disease	
Yes	Ref.
No	0.71 (0.44–1.15)

CI: confidence interval; OR: odds ratio; Ref.: Reference group.

Notes: We changed the dependent variable to a dummy variable indicating whether the respondent remained abstinent during the follow-up period (if yes = 1; otherwise = 0). The explanatory variables were the protection motivation theory component scores. We used the second generalized estimated equations to assess which protection motivation theory components were associated with sustained abstinence to further explore the reason for the higher sustained abstinence rate in the intervention group. We chose an exchangeable correlation structure for the matrix structure and binary logistic regression for the model setting.

We have not received any reports of harm or adverse eﬀects related to this intervention, for example, thumb pain while texting or any road traﬃc accidents.

## Discussion

This study joins the recent debate on mobile phone-based cessation interventions and provides some new information. The intervention group receiving personalized text messages about smoking cessation based on behaviour change theory had a 6-month quitting rate that was twice that of the intervention group getting non-personalized messages. 

We found that messages designed to counteract the intrinsic rewards of smoking was one of the important variables associated with sustained abstinence. Examples of intrinsic rewards include: “Smoking makes me feel comfortable”, “Smoking helps me concentrate” or “Smoking enhances brainwork”. In contrast, extrinsic rewards may be less important in influencing this behaviour. Examples include “Smoking looks cool and fashionable”, “Smoking is good for social networking” or “The life of a smoker is happier than that of a non-smoker”. This association has been confirmed by other studies in China.^13^^,^^19^ Therefore, encouraging smokers to correct their misperceptions of such intrinsic rewards from smoking may have better results on smoking cessation than addressing the extrinsic rewards of smoking. In addition, we found that messages focused on self-efficacy and the response cost of quitting were also associated with sustained abstinence. Self-efficacy is the belief in one’s competence to cope with adversity in specific demanding situations. A study conducted in Germany found that self-efficacy was the strongest predictor of smoking-related behavioural intention.^20^ The response cost represents beliefs about how costly performing the recommended response will be to smokers, and is significantly correlated with subsequent behaviour. This finding points to the importance of enhancing smokers’ perceived self-efficacy in quitting smoking and decreasing related response costs in further smoking cessation intervention programmes.

The comparison between groups shows that the personalized intervention decreased the intrinsic rewards of smoking and the response cost of quitting; this finding is worth noting, as both are determinants of long-term abstinence. This finding also provides some indications of why the intervention group had better results than the control group and provides further guidance for scaling up our intervention.

This study has several limitations. First, although we made efforts to ensure that both the researchers and participants remained blind to the allocation, the blinding may have been broken during multiple face-to-face contacts. Second, expired air carbon monoxide testing is the common method of detecting recent smoking and is recommended as a standard for the assessment of smoking cessation in trials. However, these biochemical tests are not perfect; expired air carbon monoxide can only be detected for approximately 24 hours after tobacco use. Third, the scale we used to evaluate protection motivation theory components was the same at each follow-up visit, and some participants may have remembered some of the questions and provided inaccurate answers. Fourth, there were only six female smokers in the cohort. Therefore, the situation among female smokers may not be fully represented when interpreting these results. Fifth, we only investigated the effects of the intervention for 6 months. We therefore do not know if anyone changed their smoking habits after 6 months. Sixth, the profile of our intervention consisted of one or two messages a day. We have no idea of what the effect would have been if a more extensive intervention were used, so we could not have a measure of what we might consider an optimal level of intervention. Seventh, some participants may live in the same community or have close contact. There was a risk of sharing messages with other participants. This could have biased our results.

Despite these limitations, our study has theoretical and policy implications. First, mobile phone-based cessation studies often suffer from insufficient reporting of the principles, and our study provides a full description of the intervention with a clear framework that can allow other researchers to take advantage of our experience. Second, we identified the psychological determinants of long-term abstinence that can allow future smoking cessation interventions to focus more on those points. However, researchers should not ignore other components of the protection motivation theory. For example, why did other variables, such as extrinsic rewards and response efficacy, show a relatively weak association with long-term abstinence? How do those variables contribute to changing smoking behaviour to achieve sustained abstinence? These questions have strong theoretical implications and need to be studied further.

Our findings suggest that the intervention we used in this study is acceptable and effective for adult smokers, without the need for additional resources, and no major challenges were encountered. It might be suitable for use in other settings with limited smoking cessation resources. The message and skills used in this intervention are not closely related to any cultural background; therefore, it might technically be easy to implement in other settings or countries with different cultural backgrounds or to target other health behaviours. The personalized evaluation tools disseminated via WeChat are also easily achieved by other online chatting tools or applications at a low cost.

This personalized text message intervention increased the rate of biochemically veriﬁed smoking cessation at 6 months. China has the world’s largest number of smokers but has inadequate resources to provide cessation services via medical institutions. If this intervention is scaled up nationwide and again demonstrated to be effective, it may benefit smokers, particularly in rural areas.
